# Unstated factors in orthopaedic decision-making: a qualitative study

**DOI:** 10.1186/1471-2474-11-213

**Published:** 2010-09-17

**Authors:** Rachael Gooberman-Hill, Anna Sansom, Caroline M Sanders, Paul A Dieppe, Jeremy Horwood, Ian D Learmonth, Susan Williams, Jenny L Donovan

**Affiliations:** 1 University of Bristol, School of Clinical Sciences, Southmead Hospital, Westbury-on-Trym, Bristol, BS10 5NB, UK; 2 University of Bristol, School of Social and Community Medicine, Canynge Hall, Whatley Road, Bristol, BS8 2PS, UK; 3 University of Manchester, National Primary Care Research and Development Centre (NPCRDC), 5th Floor, Williamson Building, Oxford Road, Manchester, M13 9PL, UK; 4 Peninsula Medical School, C420 Portland Square, Drake Circus, Plymouth, PL4 8AA, UK

## Abstract

**Background:**

Total joint replacement (TJR) of the hip or knee for osteoarthritis is among the most common elective surgical procedures. There is some inequity in provision of TJR. How decisions are made about who will have surgery may contribute to disparities in provision. The model of shared decision-making between patients and clinicians is advocated as an ideal by national bodies and guidelines. However, we do not know what happens within orthopaedic practice and whether this reflects the shared model. Our study examined how decisions are made about TJR in orthopaedic consultations.

**Methods:**

The study used a qualitative research design comprising semi-structured interviews and observations. Participants were recruited from three hospital sites and provided their time free of charge. Seven clinicians involved in decision-making about TJR were approached to take part in the study, and six agreed to do so. Seventy-seven patients due to see these clinicians about TJR were approached to take part and 26 agreed to do so. The patients' outpatient appointments ('consultations') were observed and audio-recorded. Subsequent interviews with patients and clinicians examined decisions that were made at the appointments. Data were analysed using thematic analysis.

**Results:**

Clinical and lifestyle factors were central components of the decision-making process. In addition, the roles that patients assigned to clinicians were key, as were communication styles. Patients saw clinicians as occupying expert roles and they deferred to clinicians' expertise. There was evidence that patients modified their behaviour within consultations to complement that of clinicians. Clinicians acknowledged the complexity of decision-making and provided descriptions of their own decision-making and communication styles. Patients and clinicians were aware of the use of clinical and lifestyle factors in decision-making and agreed in their description of clinicians' styles. Decisions were usually reached during consultations, but patients and clinicians sometimes said that treatment decisions had been made beforehand. Some patients expressed surprise about the decisions made in their consultations, but this did not necessarily imply dissatisfaction.

**Conclusions:**

The way in which roles and communication are played out in decision-making for TJR may affect the opportunity for shared decisions. This may contribute to variation in the provision of TJR. Making the importance of these factors explicit and highlighting the existence of patients' 'surprise' about consultation outcomes could empower patients within the decision-making process and enhance communication in orthopaedic consultations.

## Background

Total joint replacement (TJR) conducted for osteoarthritis (OA) is one of the most common elective surgical procedures. For instance, in 2008, 50,451 hip replacements and 54,709 knee replacements were conducted in England and Wales alone [1]. TJR aims to relieve chronic pain and improve function.

Patients' pathways to TJR are supported by guidance from the National Institute for Clinical Excellence (NICE) [2] in the UK, and the Osteoarthritis Research Society International (OARSI) [3]. These recommend that patients with hip or knee OA, who are not obtaining adequate management of their symptoms from non-pharmacological and pharmacological treatments, should be considered for joint replacement surgery. The National Collaborating Centre for Chronic Conditions [2] on behalf of NICE recommends that patient specific factors (including age, gender, smoking, obesity and co-morbidities) should not be barriers to referral for surgery, and that decisions on referral thresholds should be based on discussions between patients and clinicians rather than on current scoring tools. These recommendations respond to evidence that rates of TJR vary in association with age, gender, ethnicity and social class [4-15].

Research to date has explored reasons underpinning variation in provision. The parameters that clinicians usually employ include pain severity, disability and their interpretation of joint damage as seen in radiographs (x-rays). However, there is some disagreement about how these parameters may be most appropriately measured, including debate about the use of standardised scores, joint space narrowing, and the role of co-morbidities [16-19]. In practice this may lead to variation in the assessment and influence of these factors on decisions about surgery. Furthermore, various non-clinical factors may influence clinicians' decisions to recommend TJR, such as surgeons' opinions about the usefulness of joint replacement [20], their enthusiasm for TJR [21], and their beliefs about patients' preferences based on patients' gender [22].

There is evidence that patients' preferences for surgery vary in association with gender [10,23-25], ethnicity [26-30], and age [8,13,23,31,32]. The decision-making process is also influenced by how patients calculate the trade off between perceived costs and benefits [33]; their views about likely outcomes of surgery [28,34]; and their willingness to undergo surgery [32,35,36]. Furthermore, decisions about surgery incorporate patients' views about the severity of their condition and concomitant need [8,13,32] as well as their opinions about the role of clinicians [8]. Many of these factors are not easily assessed. Patients' decisions about TJR have been described as dependent on a "moving target", such that there is a constant shift in the threshold at which the balance between perceived benefits and costs tilts towards surgery [33].

In the shared decision-making model, the clinician aims to provide the medical information needed for decision-making and the patient provides information about their preferences [37]. Shared decision-making has been advocated as the ideal in TJR [38] although it is not known whether it is used, or the components of decisions within relevant orthopaedic consultations. Despite evidence that patients' preferences may vary, and that clinicians are acting on their assumptions about these preferences, we do not know how preferences and assumptions are put into action. This qualitative study explored decision-making in action within orthopaedic consultations that focused on TJR.

## Methods

### Design, setting and participants

This qualitative study comprised non-participant observation and audio-recording of consultations, followed by in-depth interviews with clinicians and patients. Data were collected in three hospital sites within two NHS Trusts in a UK city. Approvals for the study were provided by the appropriate Local Research Ethics Committee (LREC) and NHS Trust R&D offices.

Clinicians were purposively sampled to include a range of years of experience and those who specialised in hip and/or knee surgery. Seven clinicians were identified as potentially eligible and were approached to participate in the study. Four surgeons and two Extended Scope Practitioners (ESPs) gave their written consent to take part. ESPs are allied health professionals, usually physiotherapists, who have received additional training to enable them to assess and review patients in place of doctors. Clinicians consented to the researcher observing and audio-recording their consultations with consenting patients, and to taking part in both brief and in-depth audio-recorded interviews. We do not include details of surgeons' ages or other demographics because to do so in such a small sample might jeopardise their anonymity.

Patients due to see participating clinicians about the possibility of a joint replacement operation were purposively sampled to include a balance of gender, age, hip and knee OA. Seventy-seven potential participants were identified from clinic lists by NHS staff and were sent a letter of invitation and a study information booklet two weeks before their scheduled appointment. Potential participants were asked to return a reply slip to indicate if they were willing to be contacted by the researcher prior to their outpatient appointment. Of the 77 patients who were sent the booklet, 34% (26 patients) agreed to see the researcher about the study, and all of these 26 agreed to take part (free of charge). Patients gave their written consent to the researcher observing and audio-recording their consultations with clinicians, and to taking part in subsequent audio-recorded in-depth interviews.

Our team comprises researchers with interests and backgrounds in Social Anthropology, Occupational Therapy, Health Services Research, Surgery, Rheumatology, Sociology and Psychology. The study design was informed by all of these disciplines and was tailored to address the question of how decisions are made in orthopaedic practice using a design that we felt as a team would best answer this.

### Data collection

There were four stages to data collection for each clinical case:

a) The outpatient consultation for each of the 26 participating patients was observed, observational notes about the tone of interaction and setting were written and the consultations were audio-recorded. Consultations lasted between approximately 6 and 25 minutes each (26 audio-recorded observations, 26 sets of observational notes).

b) The researcher conducted a brief audio-recorded interview with the clinician immediately after the consultation once the patient had left the room. In these interviews, clinicians were asked to identify the criteria that they used in the decision-making process (24 interviews, as in two cases clinicians had urgent commitments after the consultation and were not available).

c) The researcher conducted an audio-recorded in-depth interview with each patient as soon as possible after their consultation (within three weeks). In these interviews, patients were asked to describe their pathway through care, their reflections on their outpatient appointment, including the decision that was made, the information they received, and their expectations of surgery. These interviews lasted 20-60 minutes each and were informed by a topic guide. The researcher used open-ended questions and probes to achieve detail and depth (26 audio-recorded interviews).

d) The researcher conducted an in-depth audio-recorded interview with each of the six participating clinicians. These interviews took place at various points during the study, but always after one or more of the interviewee's consultations had been observed. Interviews were scheduled to accommodate the availability of the busy clinicians and lasted up to 50 minutes each. Clinicians were asked to discuss their practice and factors that influence their decisions about offering surgery. In these interviews the researcher also used a topic guide, probes and open-ended questioning techniques (six audio-recorded interviews).

AS conducted all data collection, with the exception of two observations and two brief clinician interviews which were conducted by another member of the research team (JH) due to time constraints.

### Analysis

All audio-recordings were transcribed and anonymised. Transcripts were transferred into the qualitative analysis software package Atlas.ti. Data from the consultations and interviews were analysed together. Notes taken during observations were used to enhance interpretation of the transcribed consultations and to inform questions that were asked in interviews. Using methods of thematic analysis [39], the data were inductively coded as a whole. To achieve depth and rigour in the analysis, two team members independently read, re-read and coded a sample of the transcripts: RGH and AS coded transcripts of consultations, patient interviews and brief clinician interviews regarding three patients (9 transcripts) and the transcripts of all of the in-depth interviews with clinicians (6 transcripts). The lists of codes from the independent coding were compared with each other and a consensus about the coding frame was reached by the team. The rest of the dataset was then coded in line with this initial frame, and RG-H and AS modified and refined the coding frame as data collection and analysis progressed. Key themes were identified from the codes, cases were analysed longitudinally, idea-maps and charts of the data were produced and cases and themes were compared within and between one another using constant comparison techniques [40]. Analysis was used to inform sample size as we sought to achieve 'saturation', such that new data were not leading to any further population of the themes ('categories') with insight by the end of data collection [40]. Fieldnotes taken during observations of consultations were compared with transcripts and were used to interpret the tone and meaning of the transcribed words. As a whole, this process enabled us to identify the factors that were used in decision-making, to compare patients' and clinicians' perspectives about the 'same' decisions and to explore reasons and processes that led to decisions about treatment. Some of this data were recorded on tables for ease of data management, and material was also explored on a case basis to examine patients' journeys through decisions and care. Following this, we wrote descriptive accounts, which were developed in the context of relevant literature to arrive at theoretically informed findings [39,40]. In this article we present the findings that relate to the process of decision-making, and all names are pseudonyms.

## Results

Patients comprised 12 men and 14 women, 12 of whom had hip problems and 14 of whom had knee problems. Ages of patients ranged from 46-86 years. Details of the patients are described in Table 1. Consultation outcomes included: listing for NHS surgery; some subsequent patient decisions to opt for private surgery; alternative treatment strategies; or an offer that the patient could return for a further appointment when they felt the need (Table 2).

**Table 1:** Patients' characteristics by gender, age and affected joint (n = 26)

Age (years)	Male (n = 12)		Female (n = 14)		Total
	***Hip***	***Knee***	***Hip***	***Knee***	

< 50	0	0	1	1	**2**

50-59	1	1	0	1	**3**

60-69	3	3	2	2	**10**

70-79	0	2	3	2	**7**

80+	1	1	1	1	**4**

**Total**	**5**	**7**	**7**	**7**	**26**

**Table 2:** Patient details and consultation outcomes (n = 26)

Pseudonym	Age	Gender	Affected joint	Clinician	Outcome
Mrs Armstrong	63	Female	Right hip	Surgeon B	Listed for TJR surgery

Mrs Buckley	79	Female	Right hip	Surgeon B	Listed for TJR surgery

Mrs Crow	67	Female	Right knee	Surgeon B	Listed for TJR surgery

Mrs Darcy	63	Female	Right hip	Surgeon B	Listed for TJR surgery (opted for private)

Mrs Ellis	75	Female	Left knee	ESP 1	Listed for TJR surgery - later removed from waiting list pending further investigations of hip pain

Mrs Fox	69	Female	Left knee	ESP 1	Listed for TJR surgery (opted for private)

Mrs Glover	78	Female	Left hip	ESP 1	Not listed for TJR surgery - referred to back specialist

Mrs Holmes	48	Female	Left hip	Surgeon B	Listed for TJR surgery

Mrs Irwin	55	Female	Left knee	Surgeon D	Not listed for TJR surgery - offered open appointment

Mrs Jones	46	Female	Both knees	ESP 1	Not listed for TJR surgery - referred onward for possible trochleoplasty

Mrs Knight	77	Female	Left knee	ESP 2	Listed for TJR surgery

Mrs Lee	79	Female	Left hip	Surgeon D	Listed for TJR surgery

Mrs Miller	82	Female	Left knee	Surgeon A	Listed for TJR surgery

Mrs Norton	80	Female	Left hip	Surgeon D	Listed for TJR surgery - later declined when given surgery date

Mr Adler	77	Male	Both knees	ESP 1	Listed for TJR surgery (opted for private)

Mr Brown	64	Male	Right hip	Surgeon A	Listed for TJR surgery

Mr Campbell	79	Male	Right knee	ESP 1	Listed for TJR surgery

Mr Dutton	67	Male	Right hip	ESP 1	Listed for TJR surgery

Mr Edwards	86	Male	Left hip	Surgeon B	Listed for TJR surgery (opted for private)

Mr Franks	54	Male	Left hip	Surgeon B	Listed for TJR surgery

Mr Harris	54	Male	Both knees	Surgeon D	Not listed for TJR surgery - patient negotiated arthroscopy

Mr Ince	64	Male	Left knee	Surgeon D	Listed for TJR surgery

Mr Johnson	63	Male	Right knee	Surgeon D	Not listed for TJR surgery - referred for physiotherapy

Mr King	66	Male	Left knee	Surgeon E	Not listed for TJR surgery - advised to increase use of analgesics

Mr Lloyd	87	Male	Right knee	Surgeon E	Listed for TJR surgery

Mr Morris	66	Male	Right hip	ESP 2	Listed for TJR surgery

Clinical and lifestyle factors influenced the decisions made during outpatient consultations and were clearly described by patients and clinicians. Clinical factors were: diagnosis, symptom severity (pain and function), co-morbidities and previous treatment outcomes. Lifestyle factors were: leisure activities, work, and family life. In addition, roles ascribed to clinicians, their styles of approach and patients' adaptation to those approaches were central to the decision-making process. Roles, styles and adaptation were supported by aspects of verbal and non-verbal communication. These are discussed in turn with greatest emphasis on roles and communication, which includes description of styles and adaptation.

### Clinical Factors

Clinical factors identified by surgeons and ESPs included diagnosis of osteoarthritis and interpretation of radiography (x-ray):

Surgeon E: You'd be a good candidate in terms of the pattern of arthritis and your x-rays.

(consultation with Mr Lloyd, age 87, right knee, listed for TJR by Surgeon E)

Symptom severity, such as pain and stiffness, was assessed in considerable detail during consultations, and pain was the main indicator for TJR:

Interviewer: Tell me the main factors that you took into account with [Mr Brown]?

Surgeon A: Pain ... and restriction of walking. But principally thinking about pain.

(brief interview with clinician after consultation with Mr Brown, age 64, right hip, listed for TJR by Surgeon A)

ESP 2: Yes the main factors are restricted range of movement with pain, a lot of pain on weight bearing when she's walking.

(brief interview with clinician after consultation with Mrs Knight, age 77, left knee, listed for TJR by ESP 2)

Similarly, the presence or absence of co-morbidity and the involvement of other joints was seen as key:

ESP 1: I mean they could have a really badly arthritic hip, but equally could have dreadful feet, dreadful knees, and then awful lumber spine, and by replacing their hip isn't going to, you know, potentially be a worthwhile investment for the patient.

(in-depth interview with ESP 1)

Finally, the failure of other treatments to manage the condition was important:

Surgeon D: I mean tramadol is a pretty powerful painkiller.... So if you need that it might be an idea to do a joint replacement.

(consultation with Mrs Norton, age 80, left hip, listed for TJR by Surgeon D)

Surgeons and ESPs described clinical factors to patients during consultations. They also related them during the research interviews, providing concise case summaries:

ESP 1: The things that we generally look for are that his sleep is being disturbed on a nightly basis, he's describing a level of activity that's unacceptable to him, his pain relief is not really helping him, um and he is fit and well.

(brief interview with clinician after consultation with Mr Adler, age 77, both knees, listed for TJR by ESP 1)

Patients also reflected on the role of clinical factors in the consultations. All patients suggested that these were the main factors that clinicians took into account:

Mr Brown: I got the impression he was assessing the amount of pain I was in, the amount of movement I had, and how urgent he considered the operation to be.

(post-consultation interview, Mr Brown, age 64, right hip, listed for TJR by Surgeon A)

### Lifestyle factors

In clinicians' appraisals of suitability for surgery, they also asked patients about their preferences and needs in relation to their social and work contexts. These lifestyle factors included: leisure activities, work, and family life.

Surgeon B noted that it was appropriate for him to take lifestyle differences into account during consultations:

Surgeon B: Um and plus, you know, we're a public service, if she [Mrs Armstrong] wants to play golf and she can't play golf and I can help her to play golf, then why not?

(brief interview with clinician after consultation, Mrs Armstrong, age 63, right hip, listed for TJR by Surgeon B)

Some of the patients were in paid or voluntary work and this was identified during consultations:

ESP 1: If we take this pain away in your groin by giving you a hip replacement, what are the sort of things that you want to get back to activity-wise?

Mr Dutton: Well work.

ESP 1: What do you do?

Mr Dutton: Building.

ESP 1: You want to get back to work?

Mr Dutton: Yeah um builders never (laughs) retire, I'm afraid.

(consultation with Mr Dutton, age 67, right hip, listed for TJR ESP 1)

Clinicians also took family circumstances into account, as they were concerned to ensure appropriate support on a patient's return home following surgery:

Mrs Lee: Well the first thing he [Surgeon D] did was ask me was who lived at home. Whether I was living on my own.

(post-consultation interview, Mrs Lee, age 79, left hip, listed for TJR by Surgeon D)

### Roles and communication

In the observations and interviews it became clear that clinical and lifestyle factors were not the only elements at play in the decision-making process. Patients saw clinicians as experts, deferred to their expertise, and modified their own behaviour to complement that of clinicians. Clinicians were aware of their own decision-making styles and made judgements about their own and others' ability to conduct surgery. These roles and behaviours were supported by elements of verbal and non-verbal communication.

### Clinicians as experts

Patients saw clinicians as 'experts' and assigned the role of expert authority to clinicians during consultations. Patients deferred to clinicians' judgements:

Surgeon E: It's a judgment.

Mr Lloyd: Yeah your judgment, you're the professional.

(consultation with Mr Lloyd, age 87, right knee, listed for TJR by Surgeon E)

ESP 1: I don't feel that I would be in a huge rush to replace this hip.

Mrs Glover: No, alright then I'll do as you say.

(consultation with Mrs Glover, age 78, left hip, not listed for TJR by ESP 1)

Interviewer: Or were you going to ask for a joint replacement?

Mrs Buckley: Well no I was going to leave it entirely to him.

(post-consultation interview, age 79, right hip, listed for TJR by Surgeon B)

We explored why patients appeared to defer to clinicians' decisions and asked clinicians to describe their own views on their approaches to decision-making. Some provided a clear label for their style, for example: "paternalistic" (Surgeon A). Others described their style as they perceived it in action, for example: "diplomatic" (Surgeon B), "conservative" (Surgeon D) and "assertive" (ESP2). The clinicians' descriptions of their stance and behaviour matched those observed by the researchers, and patients also drew similar conclusions about the clinicians' styles. When particular approaches were combined with patients' perceptions that clinicians were experts, there was the potential that this influenced patients' behaviour. For instance, some patients deferred to the expert authority of their clinician even if the clinician's views contradicted their own experience. For example, Surgeon B described his style as "diplomatic" which may have influenced Mrs Darcy's choice to discount her own interpretation of the severity of her pain:

Mrs Darcy: If the surgeon had said, "Oh no it doesn't warrant having it done," well I wouldn't have had it done. You know, because I'd have thought, well it's me, you know, not coping with it well enough.

(post-consultation interview, age 63, right hip, listed for TJR by Surgeon B)

Patients' belief in the expertise of clinicians extended to patients' confidence that the clinicians were suitably skilled to perform surgery. This was an important reason for deferring to clinicians' decisions, even if a patient had not yet met their future surgeon as was the case for those who had pre-surgery consultations with an ESP rather than with an operating surgeon:

Interviewer: have you got any concerns about the surgery?

Mr Morris: Hmm I don't think so ... apparently this [Surgeon A] is the man ... so Mr [Surgeon A] is a very, very experienced hip surgeon, so you couldn't get more experienced than him anyway, so I've got full confidence in him anyway.

(post-consultation interview, age 66, right hip, listed for TJR by ESP 2)

Confidence in clinical abilities was fostered in consultations and affected how decisions were made. Patients' descriptions about confidence were echoed by clinicians' views as they expressed judgements about their own or others' abilities to conduct TJR. For instance, ESP 2 speculated that a more confident and experienced surgeon would be more willing to consider performing TJR on a patient with "lots of co-morbidities", while Surgeon D noted that his decision to offer surgery to Mrs Lee included "my assessment that I'll be able to do it without killing her."

In the majority of observed consultations the patients' and the clinicians' decision-making styles were complementary: for example, a paternalistic or authoritative clinician interacted with an apparently acquiescent or deferential patient; or a diplomatic clinician interacted with an equally diplomatic patient. This was not purely a chance finding, since there was some evidence that patients modified their behaviour to meet the style of a clinician:

Mrs Armstrong: I felt he was quite abrupt in his questioning, um and perhaps I had to be a bit quick off the mark in answering the questions.

(post-consultation interview, age 63, right hip, listed for TJR by Surgeon B)

Mr Harris: But, you know, with the consultants you can't argue. Well I can but um I don't think I'd get that far perhaps.

(post-consultation interview, age 54, both knees, not listed for TJR by Surgeon D)

### Communication

The roles assigned and adopted in consultations, and the decision-making styles and behaviours displayed, were supported by aspects of verbal and non-verbal communication.

The perception that clinicians were experts meant that some patients did not want to ask questions. For instance, Mr Franks stated that he would give control to the "specialists" and not ask "lots" of questions.

Although patients who assigned the role of expert authority to their clinicians might still internally query the decisions about proposed treatments, they would not usually express those misgivings to their clinicians:

Mr Edwards: Oh (sighs) it's not for me to question or criticise an expert opinion, but I have to express surprise that having this hip mended is going to stop all this happening.

(post-consultation interview, age 86, left hip, listed for TJR by Surgeon B)

Mr Lloyd: So all in all, I accepted his word as a professional. I thought it would be bad of me to say - to reject. And he seemed to think that, you know, that he didn't want rejection, I got that impression.

(post-consultation interview, age 87, right knee, listed for TJR by Surgeon E)

Patients viewed their relationships with clinicians and the outcomes of consultations most positively when they felt that clinicians were approachable, understandable and informative:

Mrs Buckley: It was very good. I felt that I could talk to him, you know, he was most approachable, which I thought was excellent. Because some of them, I mean they're that stiff and starchy you're frightened to open your mouth.

(post-consultation interview, age 79, right hip, listed for TJR by Surgeon B)

Patients preferred to have the opportunity to ask questions, and reflected on the rapidity of some consultations:

Interviewer: How do you feel the consultation went overall?

Mr Brown: Um I thought it was a bit quick. You know ... he may do this the next time I go there, but I would have thought he would have asked me had I any questions?

(post-consultation interview, age 64, right hip, listed for TJR by Surgeon A)

An important aspect of communication in consultations and decision-making was the use of non-verbal cues. In particular, clinicians observed non-verbal signs to support other information gathered during consultations, for example, by noting visible tiredness as evidence of night pain:

Surgeon A: Well it's [pain] - it's something they experience. Um so if you can't - I mean the most severe one is night pain, and the patient looks exhausted. Um you can tell by their faces, their faces are grey, they're drawn um, and they're haggard.

(in-depth interview with Surgeon A)

Clinicians also used non-verbal information to decide how much information to provide during consultations. For example, when asked about discussing risk with patients, Surgeon D noted that he would delay discussing risk with patients who "look terrified":

Surgeon D: If they look terrified, I'm not going to talk about it until the pre-assessment clinic when the consenting process takes place.

(in-depth interview with Surgeon D)

### The complexity of decision-making

Clinicians used consultations to appraise the information presented to them in order to reach decisions. Patients used consultations to elicit information from clinicians. Many patients were pleased with the outcome of their consultations, but some also expressed surprise about the decisions that were made at these appointments. Expressing surprise did not mean that they were necessarily dissatisfied, but indicated that the decision did not wholly match their expectations.

The complexity of surgeons' appraisals of patients' needs was identified by Surgeon A who noted that decision-making is complex, involves "various judgements", uses skills that come from "experience", and has an "instinctive" element to it:

Surgeon A: So there's various judgements that I think er are instinctive, come from experience and come from literature ... and you'll find clinicians are as good as they are old, because they've got miles on the clock, that's the key thing.

(in-depth interview with Surgeon A)

In the majority of cases, decision-making was a process that took place during the deliberations within consultations. In consultations there was usually a critical moment at which the decision to offer, or not offer, surgery was clearly stated. Deliberations that led up to this moment involved surgeons eliciting information from patients about their current status and weighing up clinical and lifestyle factors as described above. Surgeons expressed their views about patients' need through their tone as well as through literal meaning. For instance, when Mr Johnson said in his consultation that he was still able to walk around an 18-hole golf course, Surgeon D's tone of voice provided the initial indication that he considered Mr Johnson's problems not severe enough to warrant surgery:

Surgeon D: OK, how far can you walk?

Mr Johnson: If I take naproxen and paracetamols I can still get round the golf course.

Surgeon D: You can get around an 18-hole golf course?

Mr Johnson: Yeah, I might suffer afterwards.

(*consultation with Mr Johnson, age 63, right knee, not listed for TJR by Surgeon D*)

In the post-consultation interview with Mr Johnson, Mr Johnson himself also expressed awareness that his ability to walk around the golf course had been a key part of the decision not to offer him surgery:

Mr Johnson: He seemed to dismiss it [surgery] quite quickly as soon as I mentioned golf, fair enough as I say, I'm in no rush to get it done.

(*Mr Johnson, post-consultation interview, age 63, right knee, not listed for surgery by Surgeon D*)

However, in a few cases it was clear that the clinician had made some initial decisions about the appropriateness of surgery before the consultation began. For instance, immediately before the consultation with Mrs Armstrong, Surgeon B looked at her x-rays and said that in the appointment he would think about identifying the most appropriate kind of hip implant for her surgery. This indicates that he had already decided that surgery would probably be warranted for Mrs Armstrong. Patients who agreed with the offer of surgery were comfortable that some surgeons appeared to have decided that surgery was appropriate in advance of the consultations:

Mrs Holmes: But I got the impression he came in [to the consultation] and that's what he was going to do, there was no question about it. So that's fine.

(*Mrs Holmes, left hip, 48 years old, listed for TJR by Surgeon B*.

In post-consultation interviews with patients, many patients were pleased with their consultation outcomes, but others expressed misgivings. Some who were not offered surgery disagreed with the decision, but even those who had been offered surgery sometimes volunteered their surprise at other aspects of the process:

Mr Adler: I thought 'I doubt if they'll go for knee replacements before they try something less than that'. That was what I assumed. So I was, yes, I was surprised when she said [that a knee replacement was warranted]. But of course my knees have got a lot worse than what they were.

(*Mr Adler, 77 years old, both knees, listed for surgery by ESP 1, opted to have private surgery so that both knees could be replaced at the same time*)

The point at which a decision was made about surgery was clearly recognised by patients and clinicians alike. Reaching a decision about whether surgery was warranted rested on assessment of clinical and lifestyle factors, and these stated, 'explicit', factors were well recognised in the process. However, the processes through which decisions were reached were complex because of the roles that clinicians and patients played within them. The possibility that consultation outcomes might be surprising to patients was not tackled within consultations, which orientated towards making decisions rather than whether these decisions matched patients' expectations.

## Discussion and Conclusions

Agreement between clinician and patient on a course of treatment is based on the process of decision-making. The shared model of decision-making, whereby doctors and patients work together to choose the best treatment option, is recognised by the General Medical Council as a fundamental duty of doctors [41], and is the most commonly preferred style of decision-making from patients' perspectives [42]. Shared decision-making has been advocated as the ideal in TJR [38], and the Musculoskeletal Services Framework advocates patient choice, which may hinge on shared decision-making [43]. Our study has explored the process of decision-making in TJR and highlights some of the challenges of implementing shared decision-making. While decision-making in orthopaedic practice involves clinical and lifestyle indicators, roles and communication are also key. The influence of roles and communication on decisions is subtle, varies between cases, and is not described in existing guidance and recommendations.

When reflecting on their consultations, some patients later stated that they had not raised their disagreement or misgivings with surgeons and some expressed surprise about the decisions that were made. Elsewhere we address how the potential for patient dissatisfaction relates to differing perceptions between patients and clinicians about the urgency of need [44]. The findings presented here focus on the process of decision-making and the factors that are taken into account. They indicate that some patients modify their behaviour in order to better match the styles of their clinicians and that this may manifest itself as deference to the expertise of surgeons during consultations. This may be a key reason why patients do not necessarily mention any disagreement or surprise within consultations. In turn, this raises the question of the nature of a power imbalance between clinicians and patients, which may also be sustained by differential awareness of the importance of role and communication in decision-making processes. The evidence of a mismatch between clinicians' and patients' perceptions of consultations echoes findings from other secondary care settings, notably in rheumatology clinics [45]. Within orthopaedic consultations, it has been suggested that patients only raise approximately half of their concerns with their specialists [46]. Our findings provide insight into the mechanisms through which this occurs.

Furthermore, clinicians' views about their styles of practice provide important windows onto how and why patients might modify their behaviour or opinions within consultations and not raise important issues. Certain styles lend themselves to discussion and deliberation, whereas others (more 'paternalistic' ones) do not. In literature about decision-making styles it has long been suggested that there is the potential for clinicians to modify their decision-making style to suit the preferred style of individual patients [37], but it has also been shown that it is difficult to define a patient's preference purely on the basis of their communication behaviour [47]. In primary care settings it has been noted that clinicians underestimate the degree to which patients want to be involved in decision-making [48]. To achieve greater patient involvement in decision-making, our study indicates that it may be important to address the possibility that patients are modifying their behaviour to match that of clinicians. It may also be valuable to confront the issue that patients are likely to feel but not express their surprise about the outcome of their consultations. Patients may benefit from information about the unstated elements of decision-making for elective procedures (such as understanding different decision-making styles). Informing patients about these may enable them to participate more fully in shared decision-making, thus empowering patients, and meeting the requirements and recommendations of policy and guidelines [41,43].

The study's limitations included its focus on only one location and the relatively small sample of patients and clinicians. Although not ethnically representative (all participants were white) the study did include patients with a range of ages, and nearly equal representation of gender and joint type. There was also good distribution across age and experience among clinicians, although precise details are not included here in order to preserve their anonymity. Further research in this area including members of different ethnic groups would be useful. There is the potential to ask whether further variation in decision-making processes and communication may influence equity in service provision? However, a particular strength of this study was the inclusion of a number of data collection points. Interviews with clinicians immediately after observations of consultations enabled us to access their views about specific decisions. Subsequent interviews with patients up to three weeks after consultations gave patients the opportunity to reflect on the decisions and processes that occurred in their appointments. This approach appeared to enable patients to reflect on their consultations in some detail, including any misgivings or surprise. Additionally, because a researcher had been present in all consultations, there was the scope to ask questions of particular pertinence to each consultation.

The presence of the researchers (AS/JH) during consultations may have influenced the behaviour of the participants, but the researchers endeavoured to minimise this risk by being as unobtrusive as possible. Clinicians said that the presence of a researcher did not affect their practice as they were well used to the presence of others (e.g. medical students) during consultations. During the in-depth interviews, clinicians were asked to self-report on their behaviour and thought processes. It is possible there may have been some self-censoring and modifying of these reports. However, the researchers were able to evaluate whether reported behaviour matched actual behaviour during the observed consultations and were of the opinion that there was good congruence.

The findings of our study may be relevant to other elective procedures. Further research is needed to find out if the implicit elements we identified in TJR consultations are also relevant to decision-making in other conditions. Closer examination of unstated criteria used in clinical decision-making may provide greater understanding of the decision-making process and may provide subsequent opportunities to address variation and inequities in provision.

In conclusion, clinicians make judgments using unstated factors when deciding whether to offer joint replacement surgery and patients modify their behavior within consultations. If both patients and clinicians become aware of these issues and their potential influence on decision-making, then decisions about surgery could be based on a fuller range of factors. This could lead to more balanced shared decision-making and enhanced patient choice.

## Competing interests

All authors declare that they have no competing interests with regard to this study or manuscript. IL is a practicing orthopaedic surgeon.

## Authors' contributions

RGH, CS, JD, PD, JH and IL were responsible for the original study design and funding application. RGH and SW were responsible for securing ethics and regulatory approvals. AS, RGH, SW, JH and PD were responsible for recruiting participants and collecting data for the study. AS and RGH conducted the qualitative analysis of the findings. All authors were responsible for the interpretation of the findings, preparation, reading and final approval of the manuscript. RGH was the principal investigator for the project.

## Pre-publication history

The pre-publication history for this paper can be accessed here:

http://www.biomedcentral.com/1471-2474/11/213/prepub
